# Shortcuts to genetic characterization of polyposis patients in a high-risk registry

**DOI:** 10.1186/1897-4287-9-S1-O8

**Published:** 2011-03-10

**Authors:** Michelle W Done, Deborah Neklason, Randall Burt, Thérèse Tuohy

**Affiliations:** 1 Huntsman Cancer Institute, University of Utah, Salt Lake City, UT, USA; 2 Department of Oncological Sciences, University of Utah, Salt Lake City, UT, USA; 3 Department of Medicine, University of Utah, Salt Lake City, UT, USA

## Background

Mutations in the *APC* gene lead to Familial adenomatous polyposis (FAP) and an attenuated form of this condition (AFAP). Mutation detection fails using DNA-based technology in 20% of FAP and 50% of AFAP patients due to testing limitations, inability to determine significance of change, or other responsible genes. A testing protocol in our Hereditary Gastrointestinal Cancer Registry has been developed as a research tool in uncharacterized cases to rule out the obvious *APC* mutations, using a multi-step approach. This approach offers a significant cost savings which could be applied to clinical genetic testing approaches.

## Methods

FAP and AFAP cases were identified through Huntsman Cancer Institute’s Hereditary Gastrointestinal Cancer Registry. Participants were first genotyped using 4 genetic markers with high heterozygosity at the *APC* locus, spanning 2.8 Mbp. This determines if the patient may have a common ancestry with families that have known mutations. When a match was found, follow-up site-specific testing was done to confirm the known mutation. Next, two different genetic tests were applied to rule out common mutations in *APC*. The hotspot in *APC* exon 15, which accounts for 15% of *APC* mutations, was sequenced for detection of mutations between (c.3018 and c.4130). The c.426_427delAT founder mutation was evaluated through a high resolution melt-curve analysis of PCR products. CLIA- and research-negative cases were then run through an RNA-based assay that encompasses all of the *APC* intron/exon boundaries, to detect mutations that would not be found through current clinical testing methods.

## Results

Genetic markers were run on individuals from 154 kindreds, resulting in 1680 potential matches at one or both alleles at each of the 4 genetic markers. Initial analysis of the data revealed 3 ancestral mutations, and 5 coincident haplotypes; sequence verification is pending. 120 cases were analyzed for hotspot and founder mutations; 8 new hotspot and 1 founder cases were identified. 20 cases were run through RNA analysis, and 2 novel splice defects were identified. Cost analysis was run on this approach, and we find that the average cost is $500 *versus* $2500 for direct sequencing.

## Conclusions

This tiered protocol has the advantage of considerable financial savings and comprehensive evaluation of genetic changes over current clinical genetic testing approaches. The protocol is also useful for identification and expansion of missing branches of those kindreds currently enrolled in research studies and clinical trials.

